# Supplementary figures and images for: Inferring pathway activity from single-cell and spatial transcriptomics data with PaaSc
Source: PLoS Comput Biol. 2025 Nov 10;21(11):e1013666. doi: 10.1371/journal.pcbi.1013666 (PMC12622815; doi:10.1371/journal.pcbi.1013666)

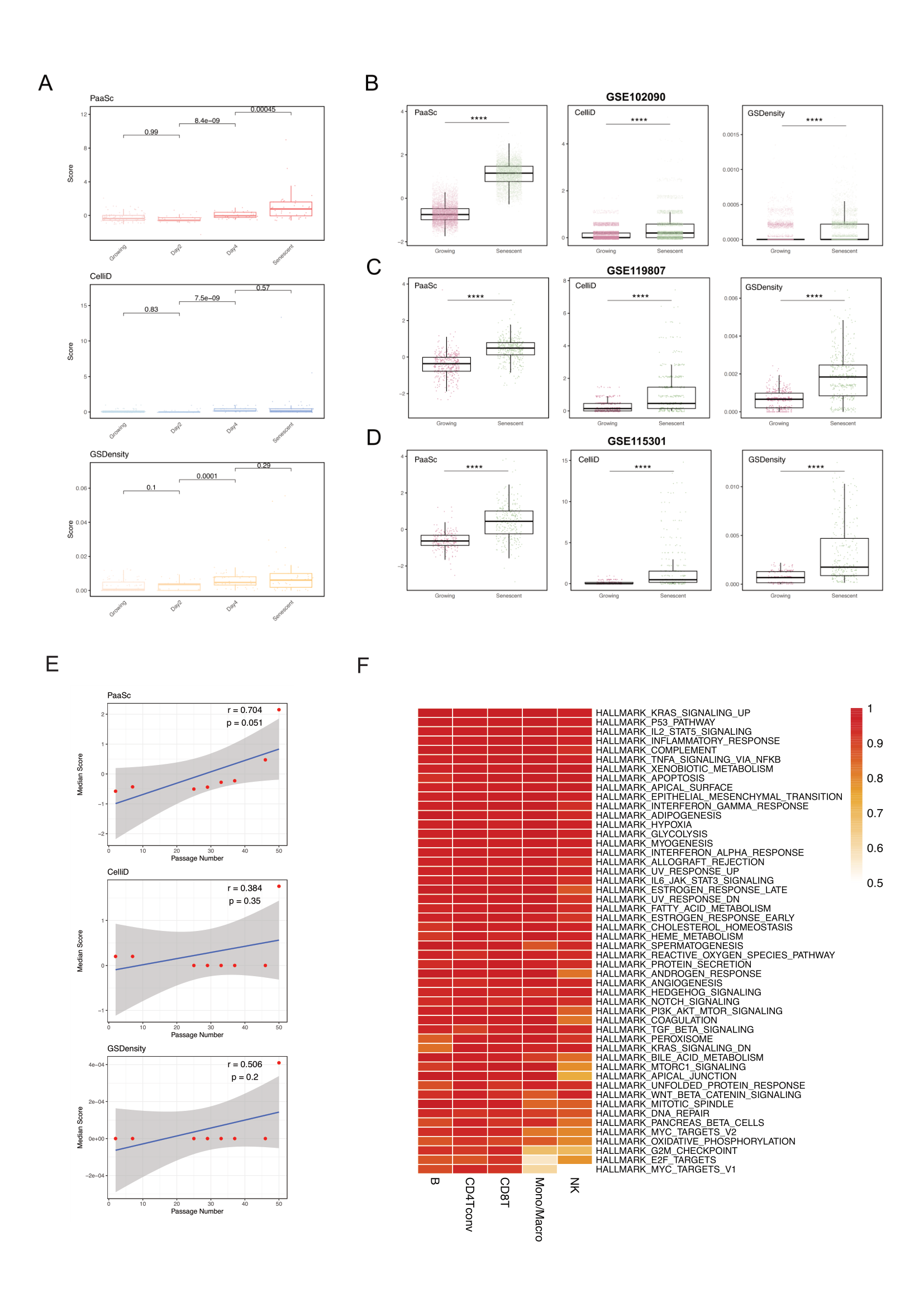

Supplement: S5 Fig — (A–D) Pathway activity score distributions calculated by PaaSc, CelliD, and GSDensity across four datasets. (E) Correlations between the median activity score and number of passages. (F) Identification of pathways associated with cell senescence by GSDensity. (TIFF) [file pcbi.1013666.s005.tiff]
