# Supplementary material for: Inferring pathway activity from single-cell and spatial transcriptomics data with PaaSc
Source: PLoS Comput Biol. 2025 Nov 10;21(11):e1013666. doi: 10.1371/journal.pcbi.1013666 (PMC12622815; doi:10.1371/journal.pcbi.1013666)
Supplement: Attachment — Submitted filename: Response-to-reviewers.docx [file pcbi.1013666.s012.docx]

**General comments to the reviewers:**

We sincerely appreciate the thoughtful and constructive feedback provided by all reviewers. In response to their valuable suggestions, we have made substantial revisions to strengthen the manuscript. The major changes are summarized below:

(**1**) Enhanced methodology presentation: We have rewritten the first section of the Results to provide a more structured and clearer description of the PaaSc methods. To further improve accessibility, we have added a new pseudocode box that illustrates the underlying algorithm step-by-step (**Figure S1**).

(**2**) Validation against batch effects: We incorporated a new figure that benchmarks PaaSc's performance for pathway activity scoring in the presence of batch effects (**Figure 6**). This analysis utilizes a public dataset profiling PBMCs from systemic lupus erythematosus (SLE) patients under two experimental conditions: control (ctrl) and interferon (IFN) stimulation (stim).

(**3**) Extension to single-cell chromatin accessibility data: We added figure to demonstrate PaaSc's versatility by investigating its application to gene set and pathway activity scoring in single-cell ATAC-seq (scATAC-seq) data, expanding the method's utility beyond traditional transcriptomic analyses (**Figure 7**).

(**4**) Spatial transcriptomics validation: We included a new figure to explore PaaSc's capability in scoring pathway activities and spatial relevance using human lung spatial transcriptomics data, thereby demonstrating its applicability to spatially resolved datasets (**Figure 9**).

(**5**) Refined discussion framework: We have reframed the Discussion section to systematically outline the key challenges in pathway activity inference and position our contributions within this broader context.

Detailed point-by-point responses to each reviewer's specific comments are provided below.

**Reviewer #1**: In the manuscript, a method called the PaaSc is proposed for inferring pathway activities for single-cell data. Benchmark comparisons with some other existing methods are done on several publicly available datasets. However, the manuscript is written in an unintelligible way, and I found it hard to comprehend the novelty of the method accurately and understand why it is better than other benchmark methods. The manuscript should be thoroughly reorganized and rewritten in a clearer and more understandable way before I can better evaluate the strengths of the proposed method.

**Response:**

We thank the reviewer for the constructive comments. In the revised manuscript, we have comprehensively rewritten the first section of the Results to provide a more structured and clearer description of the PaaSc methods. To further improve accessibility, we have added a new pseudocode box that illustrates the underlying algorithm step-by-step (**Figure S1**).

The superior performance of PaaSc is attributed to two key aspects. First, it utilizes MCA for matrix factorization, which projects both genes and cells into the same high-dimensional space, making it easier to identify nearest genes for each cell. Most importantly, PaaSc estimates the relevance of each factor and selectively uses only the most important factors for pathway activity inference, rather than utilizing all available dimensions (**Figure 1**).

We have incorporated a new figure that demonstrates this advantage by benchmarking PaaSc's performance for pathway activity scoring in the presence of batch effects. This analysis utilizes a public dataset profiling PBMCs from systemic lupus erythematosus (SLE) patients under two experimental conditions: control (ctrl) and interferon (IFN) stimulation (stim). By experimental design, cell type-specific scores should be comparable between conditions, while IFN pathway activity should be significantly higher in the stimulated condition.

The results clearly illustrate the advantage of PaaSc's selective approach (**Figure 6**). CelliD, which uses all dimensions, accurately scored cell type-specific markers (median rank normalized scores for B cells > 0.95) with no distinguishable difference between ctrl and stim conditions (AUC = 0.51, as expected). However, CelliD failed to accurately score IFN pathway activity, showing no distinguishable difference between conditions (AUC = 0.6). This occurs because the nearest genes to each cell in the high-dimensional space tend to be cell type-specific markers when all dimensions are used.

In contrast, PaaSc dynamically selects different dimensions for different pathways—cell type markers versus IFN pathway—enabling accurate scoring of both. PaaSc achieved rank normalized scores higher than 0.95 for B cell markers in both conditions and successfully distinguished IFN pathway activity in the stimulated condition, demonstrating its ability to capture pathway-specific signals while maintaining cell type resolution.

(1). The introduction of the implementation of the proposed method is confusing. The authors should present a flowchart or an Algorithm box to show how the PaaSC method works.

**Response:** We thank the reviewer for the constructive comments. We have comprehensively rewritten the first section of the Results to provide a more structured and clearer description of the PaaSc methods. To further improve accessibility, we have added a new pseudocode box that illustrates the underlying algorithm step-by-step. For convince, the new paragraph is cited below.

*The PaaSc workflow calculates pathway activity scores at the single-cell level through four sequential steps. First, cell-gene shared space construction: PaaSc employs multiple correspondence analysis (MCA) to project cells and genes from scRNA-seq data into a shared low-dimensional space. Implemented using the CelliD package [11], this process generates loading and embedding matrices. The resulting MCA representation functions as a biplot where spatial relationships—between cells, between genes, and between cells and genes—reflect their underlying associations (Fig 1A). Second, pathway-relevant dimension identification: Linear regression analysis is applied to the loading matrix to identify dimensions relevant to the pathway of interest (Fig 1B). This model evaluates whether loading factors are attributable to the target pathway versus background components. Third, dimension weight calculation: Dimensions showing significant association (P < 0.05) are retained, with their significance quantified using t-statistics—calculated as the ratio of regression coefficients to their standard errors (Fig 1C). These t-statistics serve as the primary weight for each dimension, while the proportion of variation explained by each dimension provides a secondary weight. (4) Fourth, normalized score generation: Raw pathway activity scores are computed through a weighted sum of the embedding matrix, followed by z-score normalization to standardize the scores (Fig 1D).*

*The normalized scores can be employed for various downstream analyses, including cell type annotation and testing for cluster and spatial associations. Additionally, the scores can be further binarized to classify cells into positive or negative states, assuming a bimodal distribution of pathway activity (Fig 1E). The PaaSc algorithm is further detailed in pseudocode (Figure S1) and demonstrated using the human PBMC3k dataset to assess B cell activation pathway activity at the single-cell level (Fig S2).*

(2). The organization of the manuscript is somewhat confusing. The captions should align with the figures, and I found it very difficult to go back and forth to check the figures and then jump back to the captions for illustration.

**Response**: We acknowledge the reviewer's comment regarding figure caption alignment. While we recognize this formatting issue, the journal's current submission requirements for revised manuscripts necessitate that figures and captions be submitted separately, which prevents us from addressing the alignment in this revision. We will ensure proper alignment and adherence to all formatting guidelines in future submissions.

(3). In Fig. 2, the AUC scores from different methods are compared. Why does the AUC score matter? I am not fully convinced that the proposed method performs better than the other methods. Also, it seems that GSdensity and CelliD yield a similar AUC score to the proposed PaaSC method.

**Response:** We thank the comments from the reviewer. A major challenge in evaluating pathway scoring methods is the lack of benchmark datasets. Ideally, benchmark data would contain known pathway activity measurements at single-cell resolution, but such datasets are rare. As an alternative strategy, many studies use pre-annotated cell types as ground truth and test how well cell type-specific gene set scores can distinguish target cells from other cell types. Both CelliD and GSDensity employed this benchmarking approach to evaluate their performance. In the revised manuscript, we included the following paragraph to further clarify the underlying motivation.

*Each tool generates a score for every cell that quantifies the activity of a given cell type-specific gene set. To evaluate this discriminatory capability, we constructed Receiver Operating Characteristic (AUC) curves using the pre-defined cell labels as ground truth. The Area Under the ROC Curve (AUC) served as our primary metric, measuring each tool's ability to distinguish target cell type cells (true positives) from non-target cells (true negatives).*

Our comparative analysis revealed that PaaSc and GSdensity achieved the highest performance with AUC values around 0.99, while CelliD ranked third with an AUC of 0.96 (**Figure 2B**). Despite the small difference in median AUC values, the performance gap between PaaSc/GSdensity and CelliD was statistically significant (paired t-test p-value < 0.05, Fig 2B). In summary, when benchmarking cell type-specific gene sets, PaaSc outperformed CelliD and GSdensity (**Figure 2D**), though with relatively minor AUC differences. However, when assessing real pathways, we observed larger performance differences, as detailed below.

(4). Continuing with 3, can the authors provide some theoretical justifications on why the proposed method performs better than the benchmarks? If some insightful understanding could be provided, readers would have a better understanding of the merits of the proposed method.

**Response**: PaaSc's superior performance stems from two key aspects: it uses MCA to project genes and cells into the same high-dimensional space, and most importantly, it selectively uses only the most relevant factors for each pathway rather than all available dimensions.

We added a figure to demonstrate this advantage using a public PBMC dataset from SLE patients under control and IFN-stimulated conditions (**Figure 6**). By design, cell type-specific scores should remain comparable between conditions, while IFN pathway activity should increase under stimulation. The results clearly show PaaSc's selective approach advantage. CelliD, which uses all dimensions, accurately scored cell type markers (B cell scores > 0.95) with no difference between conditions (AUC = 0.51, as expected), but failed to distinguish IFN pathway activity (AUC = 0.6). This occurs because using all dimensions biases nearest gene selection toward cell type markers. In contrast, PaaSc dynamically selects different dimensions for different pathways, achieving high B cell marker scores (> 0.95) in both conditions while successfully distinguishing IFN pathway activity under stimulation (AUC = 0.96).

We have revised the Results and Discussion sections accordingly.

(5). There are some abbreviations that make it harder to understand the manuscript. E.g., FDR, AUC, SMI... I would suggest making a list of notations at the beginning.

**Response**: We are sorry for the confusion. We have thoroughly checked and made sure all abbreviations are defined the at their appearance.

(6). Introductions on distinct datasets and their underlying biological significance will be helpful for the readers to understand better why to use those datasets.

**Response**: We thank the reviewer for the comments. A major challenge in evaluating pathway scoring methods is the lack of benchmark datasets. Ideally, benchmark data would contain known pathway activity measurements at single-cell resolution, but such datasets are rare. As an alternative strategy, many studies use pre-annotated cell types as ground truth and test how well cell type-specific gene set scores can distinguish target cells from other cell types. Both CelliD and GSDensity employed this benchmarking approach to evaluate their performance. However, cell type-specific gene sets primarily define cellular identity rather than pathway activity, making it unclear how well these tools perform when scoring biologically relevant pathways. To address this limitation, we included a dataset that profiled cells before and after interferon stimulation, providing a more biologically relevant benchmark (**Figure 6**).

(7). How are the thresholds for p and FDR determined? I noticed that different thresholds are used across different examples. Some are 0.001 while others are 0.05 or 0.0001.

**Response**: We thank the reviewer for the question. We use p-value of 0.05 as the significance level. When multiple testing is applied, we used FDR of 0.05 as the cutoff for adjusted p-values. In the manuscript, we reported the exact p-value whenever possible. However, some p-values were calculated using permutation-based method. For example, 10000 times permutation, the smallest p-value would be P < 0.0001.

Reviewer #2: The authors developed a computational method, named PaaSc, for inferring pathway activities at single cell level. The results on diverse benchmark datasets demonstrated superior performance over existing SOTA methods in multiple applications. This work has made certain improvements to the published methods. But there are several concerns should be addressed:

**Response**: We thank the positive feedback from the reviewer. In the revised manuscript, we have addressed the issues listed by the reviewer. Detailed point-by-point responses to the reviewer's specific comments are provided below.

(1) The term "pathway activities" is mentioned in the title, yet it is not directly reflected in the subtitles. It gives the impression of being a compilation of experimental results. Is it necessary to include a paragraph elaborating the association between “pathway” and the experiments?

**Response**: We thank the reviewer for the comments. A major challenge in evaluating pathway scoring methods is the lack of benchmark datasets. Ideally, benchmark data would contain known pathway activity measurements at single-cell resolution, but such datasets are rare. As an alternative strategy, many studies use pre-annotated cell types as ground truth and test how well cell type-specific gene set scores can distinguish target cells from other cell types. Both CelliD and GSDensity employed this benchmarking approach to evaluate their performance. We thus used gene-set/signature in some of the subtitles. In addition, we incorporated a new figure that benchmarks PaaSc's performance for pathway activity scoring in the presence of batch effects (**Figure 6**). This analysis utilizes a public dataset profiling PBMCs from systemic lupus erythematosus (SLE) patients under two experimental conditions: control (ctrl) and interferon (IFN) stimulation (stim).

In the revised manuscript, 4 out of 9 subtitles contains ‘pathway’: (**a**) Exploring cell senescence-associated pathways in tumor infiltrating immune cells using PaaSc. (**b**) Pathway activity scoring in the presence of batch effects. (**c**) Scoring pathway activities in scATAC-seq data. (**d**) Identification of spatial relevant pathways in lung cancer.

(2) The AUC metric in line 36 and line 90 is the same term? If the same, please maintain consistency in using the full term.

**Response**: We are sorry for the confusion. The two metrics mentioned by the reviewer are the same. the underlying algorithm are the same and were used to quantify gene-set enrichment and classification accuracy, respectively. The sentence in the Introduction now read as:

*AUCell generates cell-level gene set scores based on gene ranks within each cell using the area under the receiver operating characteristic curve (ROC).*

(3) In line 123, the name of the database should be TISCH.

**Response**: We are sorry for the typo. We have corrected this error in the revised manuscript.

(4) In line 177, “IL 16” does not appear in Figure 4E.

**Response**: We are sorry for the typo. In Figure 4E, we tested the association between activities of senescence signature and hallmark pathways from MSigDB. HALLMARK_IL6_JAK_STAT3_SIGNALING is one of the pathways that is dominated by the positive association. As described in the database website (https://www.gsea-msigdb.org/gsea/msigdb/human/geneset/HALLMARK_IL6_JAK_STAT3_SIGNALING.html), this pathway contains genes up-regulated by IL6 via STAT3. In the revised manuscript, we have use ‘IL6 JAK STAT3 signaling’ to match the term in the database and avoid confusion.

(5) In line 190, there should not be a space between “CD ” and “4”.

**Response**: We are sorry for the typo. We have corrected this error in the revised manuscript.

(6) In line 206, the middle panel should be the right panel.

**Response**: We are sorry for the typo. We have corrected this error in the revised manuscript.

(7) In line 210, the SMI approach is not described in detail in the Method section, and it's also unclear what the value of SMI represents.

**Response**: SMI represents spatial mutual information, which measure the dependence of pathway activities and spatial coordinates. For spatial transcriptomics data, PaaSc calculates pathway activity at single-cell resolution without using spatial information of each cell. Pathways were classified as spatially relevant when their activities exhibited non-random distributions across the 2D tissue section. The mathematical definition of SMI was included in the ‘**Definition and discrete approximation of spatial mutual information**’ sub-section of the Method section.

(8) In line 313, what dose the “P12M” mean?

**Response：**We are sorry for the confusion. ${\boldsymbol{P}\boldsymbol{1}}_{2M}$ refers to the sum of elements in each row of matrix $\boldsymbol{P}$, where $\mathbf{1}_{n}$ is a column vector containing n elements of 1. For matrix $\boldsymbol{P}$ with dimensions [m×n], ${\boldsymbol{P}\boldsymbol{1}}_{2M}$ for the i-th row is calculated as $\sum_{j=1}^{n} \boldsymbol{P}_{\mathrm{ij}}$, where $\boldsymbol{P}_{\mathrm{ij}}$ represents the element in the i-th row and j-th column of matrix $P$. Additionally, $\boldsymbol{1}_{\boldsymbol{N}}^{\boldsymbol{T}}\boldsymbol{P}$ denotes the sum of elements in each column of matrix $\boldsymbol{P}$. We acknowledge that the explanation of these notations was missing in the original draft. We have added the definitions in the revised manuscript.

**Reviewer #3**: Manuscript Title: Inferring pathway activities for single-cell and spatial transciptomics data with PaaSC

Summary of the Study
This manuscript addresses a major problem that is being observed across science disciplines: “translating high-dimensional data into functional pathway insights”. This manuscript really begins to address a broader problem the scientific community faces in that we generate mass quantities of data but do not fully utilize the data for discovery due to limitations in tools easily accessible or even availability. The authors developed PaaSC to address this problem with highlighting the novelty of using biologically informative components through linear regression relative to other similar models (CelliD, GSDensity) which they thoroughly benchmarked against. Through a series of rigorous computational benchmarks, the authors demonstrate that PaaSC outperforms other similar platforms in MOST scenarios. There is a need to really interrogate the processes on how data are processed for analysis. This is one area that biologist overlook not fully understanding the limitations of computational approaches in data analysis. The authors clearly understand this and have taken steps towards improving this. While the overall topic is of interest and relevant to the journal, the manuscript suffers from some minor technical, editorial, and conceptual weaknesses in its current form.

**Response:** We thank the reviewer for their positive and constructive feedback. In response to these comments, we have added three 3 new figures and substantially revised the Discussion section. These revisions significantly strengthen the manuscript's clarity and scientific rigor.

**General comments:**
1. The manuscript was well written with clearly framing the gaps in knowledge, the current state of the field, and where improvements need to be made in data analysis. This was very thoughtfully put together. The authors did very well in presenting a current limitation in analyses that have difficulties reliably identifying subtle and contextual biological changes. There is huge value in understanding subtle changes which goes to understanding cell groundstate or plasticity, an area of research more and more are accepting and going towards in our fundamental understanding of developmental biology.

**Response:** We thank the reviewer for the comments. We are pleased that the reviewer recognizes the importance of addressing current limitations in identifying subtle and contextual biological changes, which was indeed a key motivation for developing PaaSc.

The reviewer's emphasis on understanding subtle changes in cell groundstate and plasticity perfectly aligns with PaaSc's design philosophy. Rather than clustering cells into discrete groups, PaaSc enables continuous characterization of cellular states at the pathway level, making it particularly well-suited for capturing the nuanced transitions and plasticity that the reviewer highlighted. This capability is especially valuable for studying developmental processes, where cells often exist in intermediate or transitional states that traditional discrete clustering approaches may overlook.

We share the reviewer's enthusiasm for applying these approaches to developmental biology, as this field offers rich opportunities to explore cellular plasticity and state transitions. We look forward to demonstrating PaaSc's utility in developmental contexts in future work.

2. The discussion section overall read more like a summary of findings rather than a discussion. I strongly suggest the authors reframe this section, consider what their approach means, where future improvements lie and what are the major next hurdles to conquer (not just PaaSC).

**Response**: We have substantially rewritten the Discussion section to address the reviewer's concerns about it reading more like a summary than a true discussion. The revised section now provides a comprehensive analysis of what our approach means for the field, identifies major future challenges, and outlines potential solutions beyond PaaSc.

Specifically, we now discuss the broader implications of our work in the context of single-cell transcriptomics and pathway activity inference. We explain why PaaSc performs better than existing methods—through its use of Multiple Correspondence Analysis for matrix factorization and selective factor importance scoring—and what this means for advancing the field. Most importantly, we have identified and thoroughly discussed two major hurdles that the entire field must overcome: (1) the critical lack of benchmark datasets with known pathway activities at single-cell resolution, and (2) the fundamental problem of incomplete pathway definitions in existing databases, where gene sets contain pathway members rather than downstream targets. We provide concrete examples of these limitations and propose forward-looking solutions, including the use of more biologically relevant benchmarks (like interferon stimulation datasets) and emerging technologies such as Perturb-seq for better pathway modeling.

3. Reading the text and assessing the data, the language used appears to exaggerate the findings and comes off as overstating the performance. “superior” is a qualitative term and I would suggest changing this to use more quantitative measure. This will overcome that issue. For example, in line 165 the authors have done this and therefore can now use that term because they included the quantitative value.

**Response**: We thank the reviewer for the constructive comments and suggestions. We went through the main text, and for each comparison, we showed quantitative results before using qualitative terms. Briefly, we made the following changes.

(a) When describing benchmarking results in Figure 2, the following sentences were used:

*Our comparative analysis revealed that PaaSc and GSdensity achieved the highest performance with AUC values around 0.99, while CelliD ranked third with an AUC of 0.96 (Fig 2B).*

*For multiclass prediction evaluation, we compared recall, precision and F1 score which balances precision and recall. PaaSc achieving the highest median recall rate of 0.85, (Fig S3B), followed by CelliD at 0.81 (Fig 2D).*

(b) When describing benchmarking results in Figure 3, the following sentences were used:

*Among the 41 datasets containing defined B cell populations, PaaSc ranked first with a median AUC of 0.97, significantly outperforming CelliD (0.92) and VAM (0.90) (Fig 3B)*

*PaaSc achieved highest AUC scores in more datasets across all four datasets. For example, in the analysis of 92 macrophage-containing datasets, PaaSc outperformed competing methods in 36 cases (39%), while scGSEA, despite its marginally higher mean AUC score, led in only 26 cases (28%) (Fig 3E).*

(c) When describing benchmarking results in Figure 4, the following sentences were used:

*PaaSc analysis revealed progressive activation of senescence signatures (Fig 4B), showing significant differences between day 4 vs. day 2 (p = 8.4 × 10⁻⁹) and senescent cells vs. day 4 (p = 4.5 × 10⁻⁴). In contrast, CelliD and GSdensity only detected significant differences between days 4 and 2 but failed to distinguish day 4 from senescent cells (p = 0.57 and 0.29, respectively; Fig S5A).*

*To further validate these findings, we analyzed scRNA-seq data (GSE175533) [21] from WI-38 cells across sequential population doublings (PDL 20-50). PaaSc-quantified pathway activities showed strong correlation with increasing passage numbers (Pearson r = 0.7, Figure S5E). In contrast, neither CelliD nor GSdensity detected comparable progressive changes (Pearson r = 0.38 and 0.51, respectively; Fig 4C).*

(d) When describing benchmarking results in Figure 6, the following sentences were used:

*As expected, all three methods yielded AUROC values close to 0.5, indicating that cell-type-specific gene sets appropriately showed no differential activity between conditions, demonstrating robustness to batch effects. Finally, we compared the three tools' ability to detect biologically relevant pathway activity by examining IFN pathway scores. Since IFN pathways should be activated in the stimulation condition by experimental design, this serves as a positive control. PaaSc achieved the highest performance with an AUROC of 0.95, substantially outperforming CelliD (0.60) and GSdensity (0.56) (Fig 6D).*

(e) When describing benchmarking results in Figure 7, the following sentences were used:

*For each cell type, we assessed how effectively cell type-specific signature scores could distinguish target cell types from all others, as measured by AUROC. PaaSc achieved a median AUC of 0.97, outperforming both CelliD (0.93) and GSdensity (0.60) (Fig 7B). When comparing cells correctly predicted by each method, we found that predictions from PaaSc and CelliD largely overlapped, but PaaSc uniquely identified more cells (n = 1,343) compared to CelliD (n = 907) (Fig 7C).*

4. Qualitative Overreliance in the Results Section: The manuscript repeatedly uses qualitative descriptors such as “increased,” “reduced,” and “enhanced” without reporting actual fold-changes or percent differences. This weakens the impact of the findings and makes it difficult to evaluate the robustness of the effects. As the authors have done the analyses, they should include them in the narrative of the results section.

**Response**: We thank the reviewer for the constructive comments and suggestions. We went through the main text, and for each comparison, we showed quantitative results before using qualitative terms. As detailed in our response to comment 3, these comprehensive quantitative revisions have been implemented throughout the manuscript to strengthen the scientific rigor and interpretability of our results.

5. The spatial transcriptomic analysis rationale was strongly built but the outcome and what was delivered was quite underwhelming presented. This is huge area that needs to be highlighted more as there is a massive shift for people using these types of analyses. I strongly recommend the authors expand on this section. Because they spent so much time benchmarking the other data sets and approaches, they undermine themselves by not ding that here as well. Please keep the nice continuity you have built with this section and expand upon it in greater detail.

**Response**: We sincerely thank the reviewer for this valuable feedback regarding the spatial transcriptomics analysis section. We completely agree that this represents a rapidly growing and important area that deserved more comprehensive treatment in our manuscript.

In response to your recommendation, we have substantially expanded this section to include rigorous benchmarking analyses that maintain consistency with our approach throughout the paper. Specifically, we have added:

(1) Quantitative performance benchmarking: We conducted systematic comparisons of PaaSc against existing methods (GSDensity and CelliD) using annotated human brain cortical layer data, demonstrating PaaSc's superior performance with a median AUC of 0.78 versus 0.75 and 0.58 for the alternatives.
(2) Independent validation dataset: We validated our approach on a human lung cancer spatial transcriptomics dataset, where PaaSc achieved excellent cell type identification performance (median AUC = 0.97) and successfully identified spatially relevant pathways predominantly activated in tumor cells, including key cancer-related pathways such as HEDGEHOG SIGNALING, P53 PATHWAY, and PI3K AKT MTOR SIGNALING.
(3) Comprehensive spatial relevance analysis: We extended our SMI framework to systematically evaluate the spatial clustering of both cell types and biological pathways, providing quantitative measures of spatial organization that complement our pathway enrichment analyses.

These additions not only strengthen the spatial transcriptomics component but also maintain the rigorous benchmarking approach that characterizes the rest of our manuscript. We believe this expanded analysis now adequately highlights the significance and utility of spatial transcriptomics approaches while demonstrating PaaSc's robust performance in this important application domain.

6. Definitions: multiple terms are not defined in the manuscript including PaaSC, REAP-seq, etc….
**Response**: We are sorry for the confusion. We have thoroughly checked and made sure all abbreviations are defined the at their first appearance.

7. The authors discuss and analyze some biological features in Figure 4. I would suggest if they can highlight any novelty they found in these analyses that would have been overlooked by the other benchmarked approaches…this would really strengthen their arguments and convince non-computational individuals about the value of this work.

**Response**: We thank the reviewer for this valuable suggestion. To demonstrate the novelty and advantages of our approach, we benchmarked PaaSc against GSDensity, the latest method in this field, on the same 100 datasets. Notably, GSDensity could only successfully process 40 of the 100 datasets, highlighting scalability limitations. More critically, GSDensity's results showed systematic bias toward positive correlations between senescence signatures and signaling pathways (Figure S5E), which contradicts established biological knowledge. For instance, cell cycle-related pathways (E2F targets, G2M checkpoint, and MYC targets) are well-documented to be negatively correlated with senescence. While PaaSc correctly identified these expected negative correlations (Figure 4E), over 60% of GSDensity's results incorrectly showed positive correlations for these pathways.

8. Figure and Image Quality Figures general are difficult to follow. This may be a labelling problem. Color coordination patterns are mixed within figures which causes some confusion. A lot of this would be attributed to font size, scaling of the graphs, the tick marks on the axes, the labeling of the graphs. Visualization is extremely important. Figures 2 and 3 use a lot to the same box plots and when looking at this, it is too much data to take in as shown. I would suggest changing the visualization of these box blots to make it clearer what they want to show us.

**Response**: We thank the reviewer for these valuable comments on figure quality and visualization. We have addressed these concerns by comprehensively improving all figures in the revised manuscript, with particular attention to font size, color coordination, axis labeling, and overall clarity.

Most notably, we redesigned Figure 3D by replacing the dense boxplot visualization with a clearer heatmap format. The new design groups datasets by which tool achieved the highest accuracy for each cell type. For example, among 79 CD4+ T cell datasets, PaaSc achieved best performance in 33 datasets (42%), compared to scGSEA (15 datasets, 19%) and CelliD (13 datasets, 16%). This heatmap approach eliminates the visual clutter of overlapping boxplots and provides an intuitive, user-friendly comparison that clearly demonstrates PaaSc's superior performance across cell types.
