# Supplementary material for: Inferring pathway activity from single-cell and spatial transcriptomics data with PaaSc
Source: PLoS Comput Biol. 2025 Nov 10;21(11):e1013666. doi: 10.1371/journal.pcbi.1013666 (PMC12622815; doi:10.1371/journal.pcbi.1013666)
Supplement: Attachment — Submitted filename: Response-to-reviewers_auresp_2.docx [file pcbi.1013666.s013.docx]

Reviewer #1: The authors have addressed most of my concerns. However, I noticed that new typos and compiling errors were introduced. The mathematical formulas are not compiled corrected starting from Eq. 2. Therefore, I suggest that the authors check the manuscript thoroughly and revise any potential typos and compiling errors again.

**Response**:

We sincerely apologize for introducing the compiling errors and typos in the previous revision. We have carefully identified the source of these issues:

The mathematical formula compilation errors (starting from Eq. 2) were caused by software compatibility issues when converting between different versions of Microsoft Word. While the equations displayed correctly in our local Word environment, they were not properly rendered in the submission system.

We have thoroughly checked the entire manuscript to identify and correct all typos and grammatical errors. To further ensure quality, our revised manuscript has been edited for proper English language, grammar, punctuation, spelling, and overall style by highly qualified native English-speaking editors at Nature Research Editing Service (verification code: 16D7-93BB-2385-F4B8-D40P).
